# A Wavelet Adaptive Cancellation Algorithm Based on Multi-Inertial Sensors for the Reduction of Motion Artifacts in Ambulatory ECGs

**DOI:** 10.3390/s20040970

**Published:** 2020-02-11

**Authors:** Fan Xiong, Dongyi Chen, Miao Huang

**Affiliations:** School of Automation Engineering, University of Electronic Science and Technology of China, Chengdu 611731, China; xiongfan2004@163.com (F.X.); hm_93811@163.com (M.H.)

**Keywords:** adaptive cancellation algorithm, motion artifacts, inertial sensors, wavelet transform

## Abstract

Wearable electrocardiogram (ECG) devices are universally used around the world for patients who have cardiovascular disease (CVD). At present, how to suppress motion artifacts is one of the most challenging issues in the field of physiological signal processing. In this paper, we propose an adaptive cancellation algorithm based on multi-inertial sensors to suppress motion artifacts in ambulatory ECGs. Firstly, this method collects information related to the electrode motion through multi-inertial sensors. Then, the part that is not related to the electrode motion is removed through wavelet transform, which improves the correlation of the reference input signal. In this way, the ability of the adaptive cancellation algorithm to remove motion artifacts is improved in the ambulatory ECG. Subsequent experimentation demonstrated that the wavelet adaptive cancellation algorithm based on multi-inertial sensors can effectively remove motion artifacts in ambulatory ECGs.

## 1. Introduction

An electrocardiogram records a significant amount of useful information, including human heart health status. ECG is an important basis for the diagnosis of heart disease. It can also be used for diagnosing obstructive sleep apnea, evaluating therapeutic drugs, and for physiological monitoring [1]. Long-term dynamic observations of the heart can capture transient non-sustained abnormal ECG changes which are essential for diagnosing heart disease, judging curative effects, and saving lives [2]. Therefore, daily protection and long-term ECG monitoring are important methods to detect and control heart disease.

Generally, wearable ECG devices use dry electrodes that are gel-free and fabric-like, enabling long-term, dynamic, and unobstructed monitoring of ECG signals [3]. These electrodes do not make their wearers uncomfortable. However, one of most critical problems of wearable ECG devices is how to suppress motion artifacts. The difficulty in removing motion artifacts lies in their dynamic characteristics, varying frequency ranges, and relatively large amplitudes [4]. Thus, important biosignal information can be distorted or even buried. 

The current research into solving this problem is roughly divided into three directions. The first direction focuses on electrode materials and structural studies. These studies aim to eliminate motion artifacts from their sources [5,6]. The second direction focuses on signal processing via software. Principal component analysis (PCA) and independent component analysis (ICA) are used to suppress motion artifacts as independent sources of signal variations [7]. The third direction focuses on adaptive cancellation algorithms to reduce motion artifacts. This kind of algorithm uses the measurement signals of auxiliary sensors as reference input signals, including those from inertial [8], pressure [9], and photoelectric [10] sensors. 

There are several studies on suppressing motion artifacts with adaptive cancellation algorithms. Seo [11] proposed a new noise cancellation method to suppress motion artifacts. Since an adaptive filter updates its weights as it adds impulsive noise, raw ECG signals at the R wave interval are considered to be impulsive noise. Tong et al. [12] proposed an active adaptive filter for the reduction of motion artifacts. The authors designed two sensors for measuring electrode motion. These two motion sensors are a three-axis accelerometer sensor and a two-axis anisotropic magnetoresistive sensor. Pandey [13] proposed an adaptive filtering method to suppress artifacts. Reference signals were acquired by increasing the absolute power spectrum of the 3-channel accelerometer output connected to an ECG electrode. Thakor et al. [14] proposed an adaptive recurrent filter for obtaining impulse responses of the desired QRS complex. The main input signals of the adaptive filter were mixed ECG signals that contain noise. The reference input signals were an impulse train that coincided with the QRS complexes. 

The adaptive noise cancellation algorithm is currently the most widely used algorithm for the suppression of motion artifacts. An adaptive filter requires the reference signal to be highly correlated with noise in the ECG signal but remain uncorrelated with the ECG information. If the correlation is weak, the filtered output signal may be erroneous. Indeed, motion artifacts are difficult to obtain due to their time-varying features, so they are not easy to estimate in practical applications. In addition, it is difficult to find highly correlated reference signals. Instead, uncorrelated noise signals that appear in reference signals have a significant influence on the performance of the adaptive cancellation algorithm. Therefore, traditional adaptive cancellation algorithms cannot suppress motion artifacts completely.

To solve the above problems, this paper combines the wavelet transform method with the concept of data fusion to study the adaptive filter in detail. It is possible to improve the correlation by using inertial sensors installed on different parts of the body to collect more comprehensive information related to the electrode motion under different actions. Then, the uncorrelated motion information is removed by wavelet preprocessing, and the correlation of the reference input signal is further improved. This paper is organized as follows. An introduction of the data acquisition, proposed methods, and data analysis is summarized in Section 2. The results and discussion are presented in Section 3, followed by the conclusions in Section 4.

## 2. Materials and Methods

### 2.1. Data Acquisition

**Figure 1:**
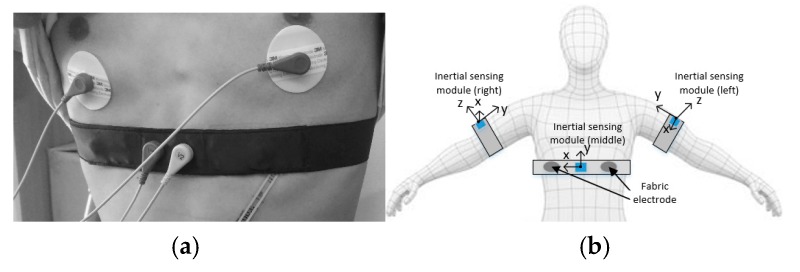
(**a**) Wearable chest strap; (**b**) inertial sensor module position.

In this paper, the wearable textile dry-electrode, shown in Figure 1a, was self-made. The placement direction of each inertial sensor module is shown in Figure 1b. In Figure 1a, the Ag/AgCl electrodes were placed near the fabric electrode. The Ag/AgCl electrodes were used to compare the signal waveform with the fabric electrode and facilitate signal synchronization. The single channel chest strap was composed of two fabric electrodes and was used to collect mixed ECG signals that contain noise signals. The mixed ECG signals were used as the main input signals for the adaptive cancellation algorithm. The inertial module (a six-axis inertial sensor MPU6050, sampling frequency 100 Hz, Bluetooth HC-06) was used to collect human motion information. Two inertial sensor modules were placed on the outside of the left and right upper arms, 8 cm from the flat shoulder. Another inertial module was placed in the middle of the two fabric electrodes. Three inertial modules were used for the acquisition of motion information, and the motion information was used as the reference input signal for the adaptive cancellation algorithm.

This experiment requires six males to wear a chest strap at a temperature of 26 °C. The chest strap is fixed at about 4 cm below the nipple with a certain initial pressure, requiring the wearer to imitate daily behaviors. ECG signals are acquired by a BIOPAC^®^ MP 150 system, with an ECG amplifier module (ECG100C). The ECG sampling frequency is 1000 Hz. Motion signals are acquired by a 6-axis inertial module. Six subjects participated, and the data were collected for 24 cases (6 subjects × 4 activities) over more than 60 s (up to 300 s).

### 2.2. Wavelet Adaptive Cancellation Algorithm

**Figure 2:**
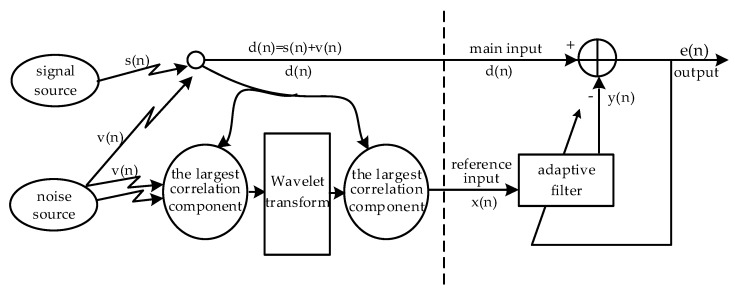
Block diagram of the wavelet adaptive cancellation algorithm.

A block diagram of the wavelet adaptive cancellation algorithm is shown in Figure 2. Firstly, the wavelet adaptive cancellation algorithm collects multipath motion signals through multi-inertial sensors. Then, we select a single motion signal that meets the requirements according to the correlation with the main input signal *d*(*n*). The main input signal *d*(*n*) consists of the ECG signal source *s*(*n*) and the unknown noise source *v*(*n*) from the subject. Hereafter, multiscale wavelet transform is performed. The desired signal *x*(*n*) is extracted as the reference input signal of the adaptive cancellation algorithm. Finally, the reference input signal *x*(*n*) is used along with the main input signal *d*(*n*) for motion artifact removal. 

The specific algorithm implementation concepts are as follows:

(1) The data sequences collected by the j^th^ inertial sensor are *X_j_*(*n*), *Y_j_*(*n*), *Z_j_*(*n*), *U_j_*(*n*), *V_j_*(*n*), and *W_j_*(*n*), which are, respectively, n-dimensional row vectors of the x, y, and z-axes, and n-dimensional row vectors around the x, y, and z-axes, where n is the number of samples, *j* = 1, 2, …, M where M is the number of inertial sensors, and the absolute value of the acceleration vector is:

(1) $$R_{j}(n)=\sqrt{X_{j}^{2}(n)+Y_{j}^{2}(n)+Z_{j}^{2}(n)}.$$

First, we calculate the correlation coefficient between the main input *d*(*n*) and *X_j_*(*n*), *Y_j_*(*n*), *Z_j_*(*n*), *U_j_*(*n*), *V_j_*(*n*), *W_j_*(*n*) and *R_j_*(*n*), We then select the component corresponding to the maximum absolute value of the correlation coefficient, recorded as *ν_j_*(*n*).

(2) First, we record the time series data collected by M inertial sensors corresponding to the largest correlation with the main input *d*(*n*) as *ν*_1_(*n*), *ν*_2_(*n*), …, *ν_M_*(*n*). We then calculate the correlation coefficients between *ν*_1_(*n*), *ν*_2_(*n*), …, *ν_j_*(*n*),…, *ν*_M_(*n*) and *d*(*n*). Finally, we select the correlation coefficient component with the largest absolute value, recorded as *ν*(*n*).

(3) By applying wavelet transform to the multiscale decomposition of *ν*(*n*), the wavelet approximation coefficients *A*_1_, *A*_2_,…, *A_L_* and wavelet detail coefficients *D*_1_, *D*_2_,…, *D_L_* [15] are obtained at different scales. The correlation between each wavelet coefficient and the main input *d*(*n*) is then calculated, removing the wavelet coefficients with little correlation. Finally, the wavelet coefficients are reconstructed according to the correlation magnitude. The wavelet coefficients with the largest absolute values are selected as the reference input signals, which are recorded as *x*(*n*).

(4) By using x as the reference input signal and d as the main input signal, the least mean square (LMS) and normalized least mean square (NLMS) adaptive cancellation algorithms are used to separate the useful ECG signal from the noise. The main steps in implementing adaptive noise cancellation based on LMS can be summarized as follows:

The first step is filtering. The filter output is given as:

(2) $$y(n)=w^{T}(n)x(n)$$

where *x*(*n*) and *w*(*n*) are n-dimensional column vectors. The order of the filter is n − 1.

The second step, error estimation, is given by:

(3) e(n)=d(n)−y(n)

The third step is to update the filter coefficients using the following equation:

(4) w(n)=w(n−1)+2μe(n)x(n)

where the parameter *μ* is a step size selected to provide convergence of the LMS adaptive filter coefficients.

The NLMS algorithm is an improved adaptive algorithm based on the LMS algorithm. The enhancement of this algorithm lies in the use of a variable step size strategy, which can shorten the time to full convergence. The corresponding weight coefficient update formula is as follows:

(5) $$w(n)=w(n-1)+[\frac{\mu }{p+x^{T}(n)x(n)}]e(n)x(n)$$

where the variable step size is:

(6) $$\mu_{e}(n)=\frac{\mu }{p+x^{T}(n)x(n)}.$$

Here, the step size is a fixed coefficient which is capable of controlling the convergence speed of the adaptive algorithms. The *p* value is usually set to a small positive number to prevent the denominator from being too small [16].

### 2.3. Data Analysis

**Figure 3:**
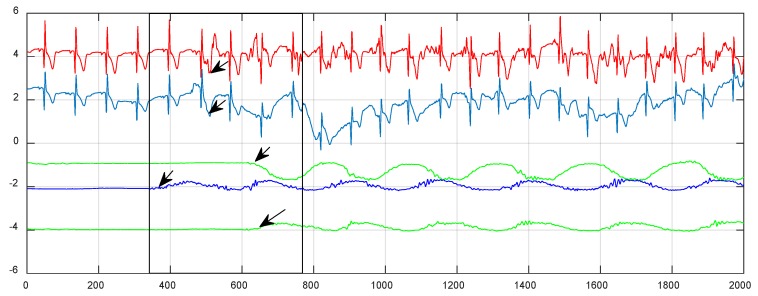
Signal synchronization (from top to bottom: Ag/AgCl electrode signal, fabric electrode signal, right, middle, and left inertial module x-axis signal).

Firstly, the reference signal is preprocessed. The sampling frequency of the inertial sensor’s motion signal is adjusted to match the ECG sampling frequency via cubic spline interpolation, which is uniformly adjusted to 200 Hz. This means that we resample the signals from the inertial sensors to 200 Hz and also downsample the ECG to 200 Hz. The inertial motion signal and the ECG signal are synchronized at the beginning of the motion. Due to the signal delay of different channels, we need to synchronize the signal manually. Figure 3 is illustrated as an example to explain the proposed synchronization method. The left side of the black arrow gives the signal collected by the subject in a stationary state. The right side of the black arrow gives the signal collected by the subject under movement. The signal represented by the black arrow indicates the moment when the subject starts to move. The Ag/AgCl electrode signal, the fabric electrode signal, and the inertial signal are aligned at the position where the black arrow points. 

Secondly, wavelet decomposition is performed with the wavelet functions sym16 and level 10; then, the wavelet coefficients of different scales are obtained. According to equation (6), we calculate the correlation between the main input signal and the motion signal collected by the inertial sensors. After that, we use the correlation to evaluate the performance of the reference signal to the adaptive cancellation algorithm, select the optimal reference signal, and eliminate motion artifacts as much as possible. The correlation coefficient between the main input signal d and the reference input signal x is defined as:

(7) $$\rho_{ds}=\frac{\mathrm{cov}(d,x)}{\sqrt{D(d)}\sqrt{D(x)}}=\frac{\sum_{i=1}^{N}\left(d_{i}-d)(x_{i}-x\right)}{\sqrt{\sum_{i=1}^{N}{\left(d_{i}-d\right)}^{2}}\sqrt{\sum_{i=1}^{N}{\left(x_{i}-x\right)}^{2}}}$$

where *ρ_ds_* is the correlation coefficient and N is the number of sampling points of the signal.

Third, in order to evaluate the performance of the reference signal for the LMS and NLMS cancellation algorithms, the signal-to-noise ratio (SNR) is calculated. A more robust approach is to use the eigenvalues of the signal autocorrelation matrix [17]. The maximum eigenvalue (*λ*_max_) represents the signal power. The sum of the remaining eigenvalues ($\sum_{n-1}^{M}\lambda_{n}$) corresponds to the noise power. The ECG signals are divided into M segments by using the wavelet transform tool [18] to calculate the eigenvalues. Each segment contains N samples of a QRS complex, forming a matrix X of M × N size. The matrix eigenvalue is given by:

(8) $$D=XX^{T}$$

The SNR is calculated as follows:

(9) $$\mathrm{SNR}=\frac{\lambda_{\max}}{\sqrt{\sum_{n-1}^{M}\lambda_{n}-\lambda_{\max}}}$$

Finally, the step size based on the LMS algorithm is set as 0.03, and the step size of the NLMS algorithm is set to 0.2. All programs and functions were written and implemented with MATLAB 2016.

## 3. Results and Discussion

**Figure 4:**
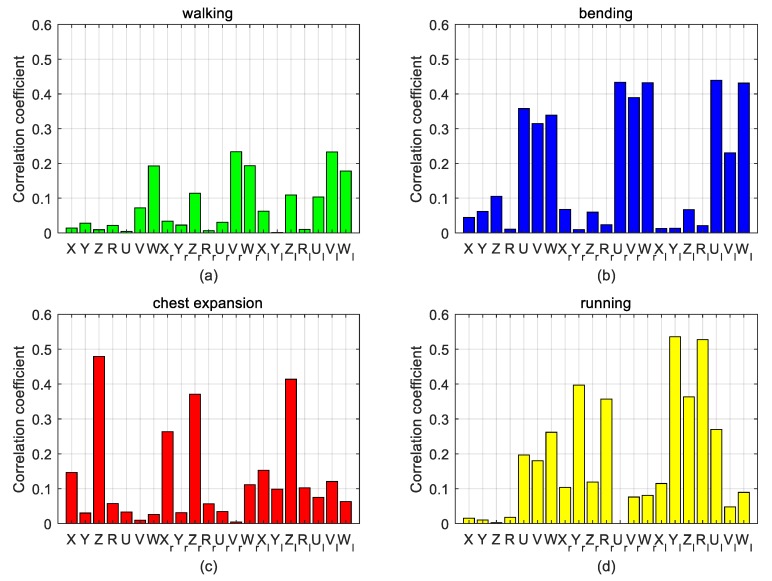
Bar graphs of the correlation coefficients.

Under daily actions (walking, bending, chest expansion, and running), the correlation coefficient between the motion signal collected by multi-inertial sensors and the ECG signal collected by the electrode is shown in Figure 4. X, Y, and Z represent the correlation coefficients between x, y, and z axis signals from the inertial sensor and ECG signals. Similarly, U, V, and W represent the correlation coefficients between the angular velocity and the ECG signal, respectively, when the gyroscope is rotated around the x, y, and z axes. R represents the correlation coefficient of the acceleration vector mode. Subscript r represents the right arm’s inertial sensors, and subscript l represents the left arm’s inertial sensors. In Figure 4, when the subject is walking, there is a strong correlation between the ECG signal and the angular velocity of the left and right arm’s inertial sensors around the y and z axes, which may be caused by the chest strap stretching. When the subject is bending, there is also a strong correlation between the ECG signal and the angular velocity of the left and right arm inertial sensors around the x and z axes. When the chest is extended, the z-axis acceleration is higher. When the subject is running, the y and z axis accelerations are highly correlated. At the same time, they are less related in a walking state due to the difficulty in creating tension in the fabric chest strap. Conversely, the correlation is stronger under the other three kinds of movements due to the tension of the fabric chest strap.

**Figure 5:**
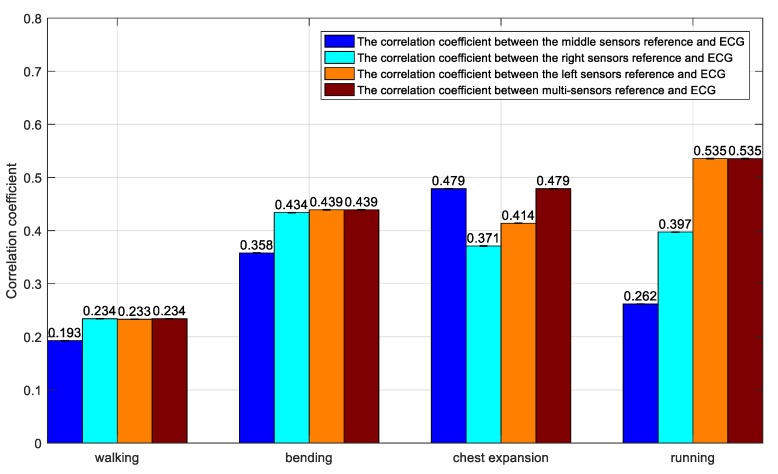
Bar graph of the absolute value of the maximum correlation coefficient.

Figure 5 shows the correlation coefficients under different states. In the walking state, if a single inertial module is used, the correlation coefficients of the three middle, right, and left positions are 0.193, 0.234, and 0.233, respectively. If multiple inertial modules are used, the correlation coefficient is 0.234. Compared to the method of using a single inertial module, the method using a multi-inertial module improved the correlation coefficient by 21%, 0%, and 0.4%, respectively.

In the bending state, if a single inertial module is used, the correlation coefficients of the three middle, right, and left positions are 0.358, 0.434, and 0.439, respectively. If multiple inertial modules are used, the correlation coefficient is 0.439. Compared with the method using a single inertial module, the method of adopting a multi-inertial module increased the correlation coefficient by 23%, 1%, and 0%, respectively.

In the chest expansion state, if a single inertial module is used, the correlation coefficients of the three middle, right, and left positions are 0.479, 0.371, and 0.414, respectively. If multiple inertial modules are used, the correlation coefficient is 0.479. Compared with the method of using a single inertial module, the method of adopting a multi-inertial module increased the mutual relationship by 0%, 11%, and 16%, respectively.

In the running state, if a single inertial module is used, the correlation coefficients of the three middle, right, and left positions are 0.262, 0.397, and 0.535, respectively. If multiple inertial modules are used, the correlation coefficient is 0.535. Compared with the method of using a single inertial module, the method of adopting a multi-inertial module increased the mutual relationship by 51%, 35%, and 0%, respectively.

Through the above analysis of daily movements, we calculated the correlation between the motion signals collected by single inertial sensors and multi-inertial sensors and the ECG signals collected by the electrodes. It can be concluded that the reference signal of the adaptive motion artifact cancellation algorithm based on the inertial sensors is not only related to the position where the inertial sensor is placed but is also related to the multiple inertial sensors distributed on different parts of the body. Using multiple inertial sensors is more efficient as they allow one to sense more correlations with motion artifacts in the ECG.

**Figure 6:**
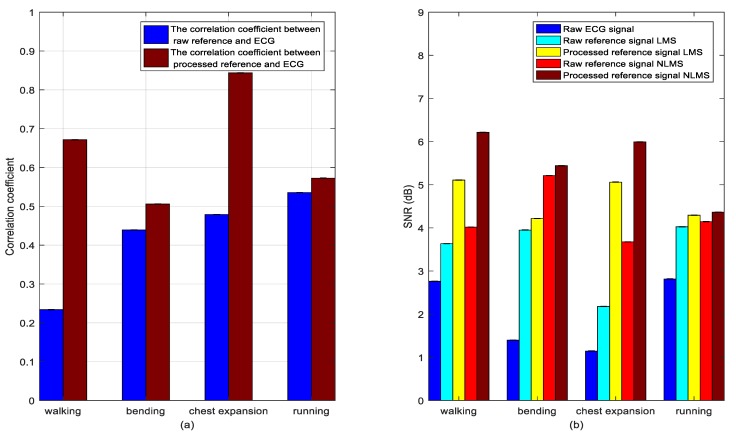
(**a**) Correlation coefficients between reference signal and main input ECG signal before and after wavelet transform preprocessing and (**b**) SNR before and after filtering.

Under different actions, the measured components corresponding to the maximum correlation coefficient values of the three inertial sensors were selected. Multiscale wavelet decomposition is used to preserve the wavelet coefficients with large correlation coefficients to reconstruct the signals. The component corresponding to the maximum correlation coefficient is then selected as the reference input signal. Figure 6a shows the correlation coefficient values (the absolute values) before and after the wavelet transform processing reference signal, and Figure 6b shows the SNR under several different states. Obviously, the correlation coefficient and SNR are improved to different degrees under different actions.

The data from Table 1 show that the correlation improvement is greater after wavelet preprocessing in the case of walking and chest expansions, increasing from 0.2340 to 0.6716 and from 0.4789 to 0.8437, respectively. Under different actions (walking, bending, chest expansion, and running), the SNR is also correspondingly improved to varying degrees after applying the LMS and NLMS adaptive cancellation algorithms, as shown in Figure 6b. This can also be seen from the data in Table 1; after applying the LMS algorithm under different actions, the SNR increased from 3.6346, 3.9511, 2.1802, and 4.0256 to 5.1084, 4.2186, 5.0617, and 4.2943. After applying the NLMS algorithm, the SNR under different actions increased from 4.0197, 5.2128, 3.6769, and 4.1449 to 6.2146, 5.4424, 5.9939, and 4.3650, respectively.

**Figure 7:**
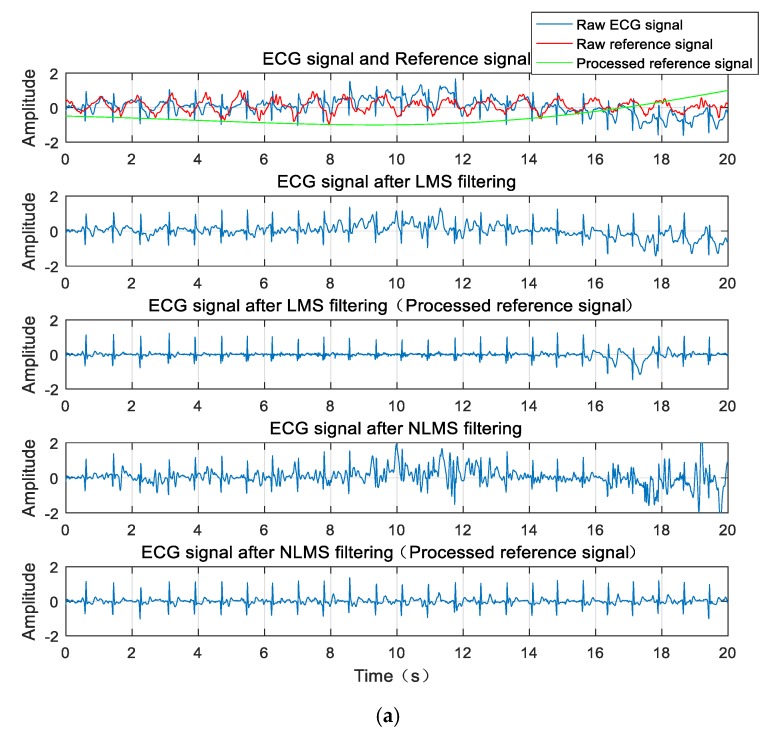

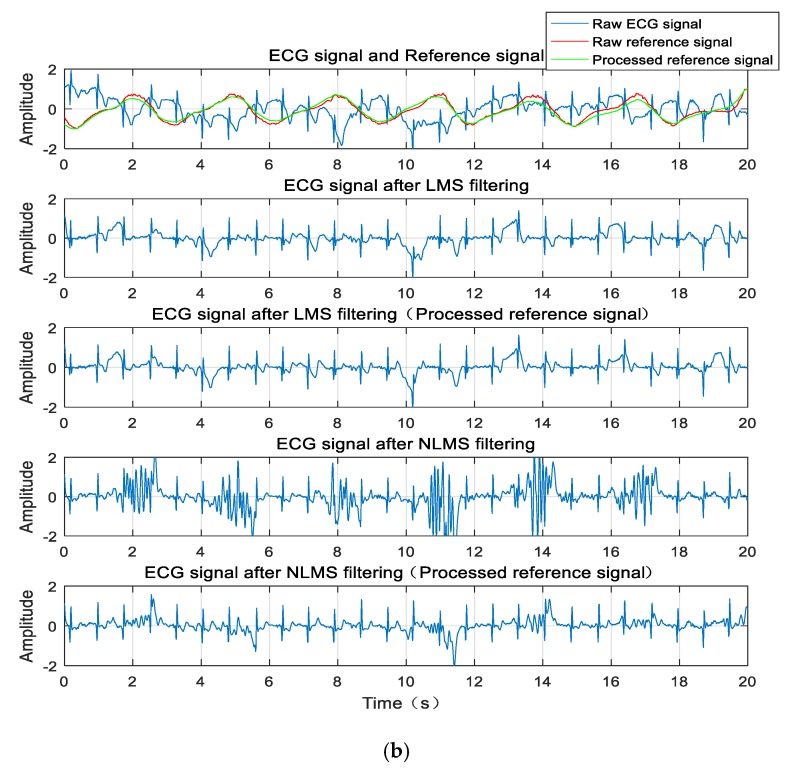
ECG signal before filtering, the reference signal, and the filtered ECG signal waveforms for (**a**) walking and (**b**) bending.

The waveform diagram is shown in Figure 7. Compared to the reference signal (red line) before wavelet transform processing, the reference signal (green line) processed by the wavelet transform is more similar to the original ECG signal. Correspondingly, these two filtering methods are compared before and after. The ECG signals produced by the adaptive cancellation algorithm after wavelet transform processing all have different degrees of improvement. The waveform improvement is most obvious in the walking state of the subject, where the correlation is greatly improved. The QRS complex is also apparent. With the exception of a few waveforms, the P and T waves are also visible.

In summary, the data collected by the inertial sensors installed on different parts of the body are different under different actions. The multiple inertial values compensate for the lack of relative motion information from the single inertial sensors, and the correlation of the reference input signal is improved in the adaptive cancellation algorithm.

The reference signal is decomposed by the multiscale wavelet, removing the uncorrelated wavelet coefficients. The high wavelet correlations are retained to reconstruct the reference input signal. This method increases the correlation effectively. The SNR of the ECG signal is improved after the adaptive cancellation algorithm, and the ECG waveform is also improved. This occurs because the inertial sensors are mounted on the outside of the clothes, and the fabric electrodes are in close contact with the surface of the human skin. Therefore, when the human body moves, the inertial sensors inevitably create vibrations, and the electrodes may not be displaced or vibrated. At this time, the inertia generates motion information that is not related to the electrode artifacts. If such a motion signal is used as the reference input signal for the adaptive cancellation algorithm, additional noise will be introduced, resulting in signal distortion, while the wavelet decomposition can remove wavelet coefficients with little or no correlation. The signal is then reconstructed with the preserved wavelet coefficients, which is equivalent to removing a portion of the uncorrelated motion information. In this way, the correlation of the reference input signal is increased. The above results only show the data of one participant. The test results of the other five participants have similar rules and conclusions.

## 4. Conclusions

In this paper, we adopted self-made wearable textile dry-electrode chest belts to collect ECG signals. Motion information was collected through inertial sensors installed on different parts of the body. By adding inertial sensors, more comprehensive motion information can be collected, allowing the motion information to be more accurately predicted and recorded. We then used wavelet transform to remove irrelevant motion information to further improve the correlation of the reference signal. The experimental results show that the wavelet adaptive cancellation algorithm based on multi-inertial sensors can more effectively suppress motion artifacts in dynamic ECGs.

## Figures and Tables

**Table 1:** SNR before and after filtering based on the LMS and NLMS algorithms for different motion signal inputs.

	Reference Signal Before Wavelet Preprocessing				Reference Signal After Wavelet Preprocessing			
Motions	Walking	Bending	Chest Expansion	Running	Walking	Bending	Chest Expansion	Running
Correlation	0.2340	0.4391	0.4789	0.5355	0.6716	0.5059	0.8437	0.5727
SNR before filtering	2.7628	1.3974	1.1470	2.8169	2.7628	1.3974	1.1470	2.8169
SNR after filtering by LMS	3.6346	3.9511	2.1802	4.0256	5.1084	4.2186	5.0617	4.2943
SNR after filtering by NLMS	4.0197	5.2128	3.6769	4.1449	6.2146	5.4424	5.9939	4.3650
